# Analysis of lower urinary tract signs and bacteriuria in cats with subcutaneous ureteral bypass systems

**DOI:** 10.1002/vro2.69

**Published:** 2023-08-01

**Authors:** Luba Djoneva, Jack Lawson, Lynda Rutherford, Rebecca Geddes

**Affiliations:** ^1^ Royal Veterinary College North Mymms Hatfield Hertfordshire UK

## Abstract

**Objectives:**

Distinguishing bacterial cystitis from subclinical bacteriuria is necessary for feline treatment protocols and antimicrobial stewardship. This can be challenging in cats with subcutaneous ureteral bypass (SUB) systems because they may present with lower urinary tract signs without bacteriuria. We investigated the relationship between positive urine culture and lower urinary tract signs in cats with SUBs, including factors associated with each.

**Methods:**

Clinical records were retrospectively reviewed to identify cats presenting with ureteral obstruction that underwent placement of a SUB device(s). The relationship between a positive urine culture and lower urinary tract signs was determined by chi‐squared analysis. Univariable and multivariable logistic regression models were performed to identify factors associated with positive urine cultures and lower urinary tract signs.

**Results:**

Two hundred and thirty visits were recorded for 61 cats, with 36 of 230 (16%) positive cultures in 21 of 61 (34%) cats. Lower urinary tract signs were documented at 97 of 230 (42%) visits, with 37 of 61 (61%) cats demonstrating lower urinary tract signs at some point. No relationship was found between culture results and lower urinary tract signs. Risk factors for a positive culture were higher urine pH, higher urine white blood cells and the presence of bacteriuria on microscopy. Risk factors for lower urinary tract signs were younger age and being a purebred cat (vs. non‐purebred).

**Conclusions:**

A high proportion of cats with SUBs exhibited sterile lower urinary tract signs, making differentiation between bacterial cystitis and subclinical bacteriuria difficult. This highlights the need for clearer guidelines on when to treat bacteriuria in cats with SUBs.

## INTRODUCTION

Ureteral obstructions are common in cats, with the majority caused by uroliths, although other causes include ureteral stricture, blood clots, neoplasia, trauma and iatrogenic injury.^1^ Obstruction can cause acute kidney injury and treatment is necessary to allow for renal decompression and return of renal function. Obstruction can be relieved by medical management, although this is frequently unsuccessful and surgical intervention is required in the majority of cats.^2^ Ureterotomy, ureterectomy and ureteral stent placement have historically been associated with up to 31% major complication rates and mortality at discharge up to 21%.^2^, ^3^ Subcutaneous ureteral bypass (SUB) systems are an alternative treatment method that consists of a nephrostomy catheter in the renal pelvis and a cystostomy catheter in the bladder lumen that are connected to a subcutaneous port.^4^ This allows urine to flow from the kidney to the bladder, bypassing the obstructed ureter and allowing renal decompression and function to return. Subcutaneous ureteral bypass allows preservation of the affected kidney and typically has a lower mortality rate of 5%–12%.^4^, ^5^, ^6^, ^7^, ^8^, ^9^

A common complication of SUB placement is urinary tract infection (UTI), with bacteriuria found at some timepoint in 24%–54% of cats following SUB placement.^4^, ^5^, ^6^, ^7^, ^8^, ^10^, ^11^, ^12^ A UTI is thought to account for 43%–93% of positive culture results, with the remainder representing subclinical bacteriuria (SB).^6^, ^7^, ^10^, ^11^ Most commonly UTIs appear confined to the lower urinary tract despite the presence of a SUB; therefore, ‘bacterial cystitis’ (BC) is more appropriate terminology. The presence of one or more lower urinary tract signs (LUTS) (haematuria, stranguria, dysuria, pollakiuria) differentiates BC from SB, with SB defined as a positive urine culture in the absence of clinical signs.^13^

Previous studies have reported that 3.5%–18% of cats with SUBs exhibit LUTS despite a negative culture,^4^, ^5^, ^8^ with the LUTS thought to be due to the irritation caused by the cystostomy catheter.^14^ Therefore, there is likely to be difficulty in distinguishing BC from SB in cats with SUBs because some cats show LUTS despite negative urine culture. However, this distinction is important because current guidelines suggest that SB should not be treated, although management of SB in cats with urinary tract implants is not specifically discussed.^13^ Bacterial cystitis is one of the most common reasons for using antimicrobials in veterinary practice,^13^, ^15^ so differentiation should be made between BC and SB to decrease antimicrobial use and limit antimicrobial resistance.^16^ Studies have looked at the incidence of UTI and SB in cats with SUBs^4^, ^5^, ^6^, ^7^, ^8^, ^9^, ^10^, ^11^; however, detailed information describing the presence of LUTS and the concurrent incidence of sterile cystitis is lacking.

We hypothesised that the presence of a SUB(s) masks the ability of clinicians to differentiate BC from SB in cats. This study therefore aimed to document the incidence of and assess the correlation between LUTS and positive urine culture in cats with SUBs and to further determine factors that were associated with either a positive urine culture or the presence of LUTS in these cats.

## MATERIALS AND METHODS

### Data collection

The electronic records of all cats that had a SUB system or systems implanted at the Queen Mother Hospital for Animals (QMHA) between January 2019 and March 2022 with at least one follow‐up visit that involved a urine culture were retrospectively reviewed.

Data recorded from the initial visit when SUB placement surgery was performed included signalment, surgical factors (length of hospitalisation and bilateral vs. unilateral SUB placement) and urine culture results. Lower urinary tract signs were rarely recorded at the initial visit and were therefore not investigated. Cats were separated into purebred and non‐purebred categories, where any cases recorded as domestic short, medium or long hair were ‘non‐purebred’ and all other breeds were ‘purebred’. The aetiology of ureteral obstruction was determined based on ultrasound, radiographs and/or surgical findings.

Follow‐up visits where a urine culture was performed were included up until 14 March 2022. The date and elapsed time since the previous visit were noted. Owners completed a standardised history questionnaire at each re‐examination visit to the QMHA following their cats’ SUB(s) placement (see Supporting Information S1). The completed questionnaire and the attending clinician's written report were reviewed with an emphasis on LUTS and medication. Lower urinary tract signs were defined as gross haematuria, stranguria and/or pollakiuria. Pollakiuria was defined as abnormally frequent urination as reported by the owner for their cat or as more than six urinations per day based on a mean of 2.1 urinations per day in healthy cats.^17^ Drinking volume and owner report of quantity of urine were utilised to distinguish between pollakiuria and polyuria. If signs were noted to have been transient and resolved prior to re‐examination, ‘no LUTS’ was recorded for that visit. However, if signs were noted to be intermittent or to have commenced immediately prior to the visit, the cat was classified as having LUTS at that timepoint. If owners did not report any LUTS or they were ‘unknown’, it was assumed that signs were not present.

Urine samples were submitted for culture, urine‐specific gravity (USG), dipstick and microscopic examination. When multiple urine samples were taken during the same visit, results from urine samples obtained in a sterile manner via the SUB port(s) were used for urinalysis and culture. Cultures were considered positive if any growth was found in samples from SUB ports or cystocentesis and if greater than 100,000 colony forming unit/mL bacterial growth was found in free catch samples, as per International Society for Companion Animal Infectious Diseases (ISCAID) guidelines.^13^ In cases with bilateral SUBs, if urinalysis was performed separately on both sides, the most abnormal result between both for each category was used for analysis. Urinalysis results were categorised for analysis (see Supporting Information S2). Microscopic haematuria and pyuria were defined as more than 10 red blood cells (RBCs) per high power field (HPF) and more than five white blood cells (WBCs) per HPF, respectively.^5^, ^18^, ^19^, ^20^, ^21^ Active sediment was defined as haematuria, pyuria and/or bacteriuria.^18^, ^19^

Blood analysis, non‐invasive blood pressure (by Doppler) and body condition score were noted when available. Manual packed cell volume and total solids were preferentially used for analysis over machine readings. Sodium, potassium, chloride, urea, creatinine and ionised calcium were measured most frequently using an iSTAT (Abaxis VetScan 300 V, s/n 705971), occasionally on a Radiometer ABL800 Flex Analyser and/or included on a biochemistry panel. When multiple blood samples were evaluated, the first sample of a visit was used and iSTAT was used preferentially. Inorganic phosphorus was measured at the Royal Veterinary College Diagnostics Laboratory.

### Statistical analyses

To make data independent for statistical analysis, only one visit was utilised for each cat. If a positive culture was recorded for a cat, the first positive culture visit was used. Improvements in creatinine can be seen for weeks to months following SUB placement^9^, ^22^; therefore, to allow for creatinine to plateau prior to inclusion in statistical models and to allow for postoperative healing prior to assessment of LUTS, the visit selected for cats that always had a negative culture was as follows: the first visit more than 2 months following SUB(s) placement with urinalysis results documented from a sample taken via the SUB port and all recorded blood parameters was used, or the first visit more than 2 months following SUB(s) placement if no visit had all data available for review. Medication was reviewed to ensure that the cat was not receiving antibiotics during this included visit. Continuous variables were reported as medians and ranges for negative and positive cultures separately and compared between groups using a Mann–Whitney *U*‐test.

A chi‐squared test for independence was performed comparing culture results (positive or negative) and LUTS (present or absent).

R (version 4.2.1, R Foundation for Statistical Computing, www.R‐project.org) was used to run logistic regression analysis. Two univariable logistic regression models were performed to examine risk factors associated with (a) positive (outcome 1) versus negative (outcome 0) urine culture results and (b) the presence (outcome 1) versus absence (outcome 0) of LUTS. Factors that were significant in univariable analyses at *p*‐values less than 0.10 were included in the two multivariable models, where backward elimination was used until only variables with *p*‐values less than 0.05 remained. Model assumptions were checked by assessing correlations between independent variables, linearity of continuous variables against the log odds of the dependent variable and the presence of outliers using Cook's distance values. The Hosmer–Lemeshow test was used to assess goodness of fit.

All statistical tests (chi‐squared analysis, logistic regression, Mann–Whitney *U*‐tests) were based on one visit per cat and were evaluated at 0.05 significance.

## RESULTS

Seventy‐three cats had SUB system(s) placed at the QMHA between January 2019 and February 2022. Of these, 12 were excluded from analyses due to having no follow‐up visits at the QMHA after initial SUB placement, resulting in 61 cats being included in this study.

Of these 61 cats, 36 were spayed females (59%), 23 were neutered males (37%) and one each (1.6%) was an entire female or entire male. The median age at the time of SUB(s) placement was 7.4 years (0.4–15 years). Cats were described as domestic short hair (32; 52%), British short hair (6; 9.8%), Ragdoll (6; 9.8%), domestic long hair (4; 6.6%), Australian mist (2; 3.3%), Birman (2; 3.3%) and one of each of the following breeds (1.6%): Bengal‐Somali cross, Brazilian short hair, British blue, British long hair, Domestic medium hair, Don Sphynx, Persian, Sphynx and Turkish Van. Therefore, 37 of 61 (61%) were considered non‐purebred and 24 of 61 (39%) were purebred.

Ureteral obstruction was due to urolithiasis (55; 90%), stricture (2; 3.3%), blood clot (1; 1.6%), iatrogenic injury of ureter during spay surgery (1; 1.6%) or an unknown reason but unlikely to be a urolith (2; 3.3%). Subcutaneous ureteral bypass placement was either unilateral (22 each side; 72%) or bilateral (17; 28%). At the time of surgery, urine culture results were positive in 11 (18%) cats and negative in 50 (82%) cats. The median duration of hospitalisation for the SUB(s) placement visit was 8 days (5–24 days).

For the 61 cats included, 230 follow‐up visits following SUB(s) placement were recorded. Of the 230 visits, LUTS were reported at 97 (42%) visits, with pollakiuria reported most frequently at 74 (32%) visits, followed by stranguria (71; 31%) and gross haematuria (40; 17%). Gross haematuria was only reported as the sole LUTS at two of 230 visits. Twenty‐four cats never showed any LUTS (39%), with the remaining 37 of 61 (61%) cats having LUTS reported on at least one visit. Lower urinary tract signs were reported at 81 of 230 (35%) visits despite a negative culture.

A positive urine culture was documented at 36 of 230 (16%) visits, occurring in 21 of 61 (34%) cats. Of these 21 cats, 15 (71%) were female spayed, five (24%) were male neutered and one (5%) was female entire.

Of the 21 cats that demonstrated at least one positive culture during follow‐up, five (24%; 8% total cohort) were diagnosed with BC based on the presence of concurrent LUTS at the time of a positive urine culture and an absence of LUTS at other time points when urine culture was negative. Of 21 cats, SB was diagnosed in 10 (48%; 16% total cohort) cats due to an absence of LUTS at the time of a positive urine culture and the remaining six (29%; 10% total cohort) cats demonstrated inconsistency between the presence of LUTS and the concurrent presence of a positive urine culture making differentiation between BC and SB impossible.

Of the 36 positive cultures, 16 (44%) had concurrent LUTS and 20 (56%) occurred in the absence of LUTS. Table 1 shows which bacteria were cultured and if LUTS were present. Five cultures (four cats) cultured two bacterial species; the remaining were all single species. One cat had a mixed infection of *Escherichia coli* and *Enterococcus faecalis*, and this cat did not show clinical signs. Two cats urine samples cultured for multidrug‐resistant bacteria, one *E. faecalis* (three occasions) and one *Klebsiella pneumoniae* and *Pseudomonas aeruginosa* (two occasions). Ten cats had more than one positive culture of the same bacterial species, with one of these 10 cats demonstrating recurrence of the bacteria as opposed to persistence (three negative urine cultures off antibiotics between positive urine cultures).

**TABLE 1:** Bacteria cultured and how frequently in cats with subcutaneous ureteral bypass.

Bacteria	Number of cultures	Number of cats	Lower urinary tract signs
*Escherichia coli*	22	12^a^	Yes—13 cultures (four cats only with positive cultures, four cats inconsistently) No—nine cultures (four cats)
*Enterobacter cloacae*	7	4	No
*Staphylococcus pasteuri*	2	1	No
*Serratia ureilytica*, *Serratia marcescens*	1	1	Yes—only with positive cultures
*Enterobacter cloacae*	1	1	No
*Staphylococcus pasteuri*, *Psychrobacter faecalis*	1	1	No
*Klebsiella pneumoniae*, *Pseudomonas aeruginosa*	2	1	Signs on one visit, no signs on other visit
Gram‐positive bacilli	1	1	Yes—always showed signs regardless of culture

*Note*: One cat occurs twice because it had a mixed infection of *E. coli* and *E. faecalis*, it did not show clinical signs.

^a^ Two cats had two separate positive cultures.

Urine sediment examination was performed on 218 samples, of which 209 (96%) demonstrated an active sediment: 186 (85%) had microscopic haematuria, 177 (81%) had pyuria and 35 (16%) had bacteriuria. Bacteria was noted on sediment examination in 22 of 36 (61%) positive cultures.

A comparison of clinicopathological data between cats with a positive urine culture result and a single equivalent timepoint for a negative urine culture result is shown in Table 2. The time elapsed between study visit and date of SUB(s) placement was not significantly different between positive and negative cultures (negative cultures 118 days [22–415 days] vs. positive cultures 54 days [11–642 days], *p* = 0.280). Cats with a positive urine culture had higher urinary pH measurements but lower USG measurements than cats with negative urine cultures (positive cultures pH 6 [5–8] vs. negative cultures pH 5.5 [3–8], *p* = 0.003; positive cultures USG 1.017 [1.010–1.030] vs. negative cultures USG 1.021 [1.011–1.030], *p* = 0.032). Hospitalisation length was not significantly different (*p* = 0.197) between cats with positive and negative cultures despite all five cats with hospitalisation visits over 2 weeks subsequently having a positive culture. Overall, no association was found between a positive culture result and the presence of LUTS (*p* = 0.301).

**TABLE 2:** Clinicopathological data for negative and positive bacterial cultures with subcutaneous ureteral bypass (SUB), with corresponding Mann–Whitney *U*‐test *p*‐value.

Variable	Unit	Negative culture (*n* = 40)		Positive culture (*n* = 21)		*p*‐Value
		Median	Range	Median	Range	
Time elapsed from surgery to visit date	Days	118	22–415	54	11–642	0.280
Hospitalisation during SUB placement	Days	8	5–13	9	5–24	0.197
Age at visit	Years	7.97	0.9–15	8.1	0.4–14.25	0.964
Time from previous flush	Months	3	0.5–9	1	0.5–7	0.080
Urine‐specific gravity	×1000	1021	1011−1035	1017	1010−1030	0.032^a^
pH		5.5	3–8	6	5–8	0.003^a^
Packed cell volume	%	32	17–51	28	18–45	0.115
Total solids	g/L	74.5	59–93	72.5	53–91	0.880
Sodium	mmol/L	152	132–157	152	145−159	0.782
Potassium	mmol/L	3.8	2–5.9	4	2–6.8	0.215
Chloride	mmol/L	121	101–133	121	93–127	0.339
Inorganic phosphorus	mmol/L	1.44	0.86–3.81	1.57	1.05–3.06	0.173
Urea	mmol/L	14.1	7.6–43.9	14.8	7.9–50	0.920
Creatinine	µmol/L	178	86−539.2	195	68–621	0.236
Ionised calcium	mmol/L	1.38	1.18–1.82	1.38	1.18–1.61	0.584
Blood pressure	mmHg	138	75–200	127.5	90–150	0.427

^a^ Significant values.

Univariable and multivariable logistic regression model results for risk factors associated with a positive urine culture result are depicted in Table 3. Two visits were excluded from the multivariable analysis due to missing urinalyses. Multivariable analysis identified urine pH (odds ratio [OR] 3.18, 95% confidence interval [CI] 1.04–12.13), marked pyuria (greater than 50 WBCs/HPF when compared to less than 5 WBCs/HPF; OR 157.68, 95% CI 7.71–25,987.11) and bacteria on sediment (OR 21.71, 95% CI 2.33–588.10) to be independent predictors of a positive urine culture. The model was a good fit (Hosmer–Lemeshow, *p* = 0.973).

**TABLE 3:** Logistic regression outcomes for positive urine cultures in cats with subcutaneous ureteral bypass (SUB).

Variable	*p*‐Value	Options	Odds ratio	95% confidence interval
**Univariable analysis**				
*Cat and surgery factors*				
Type of surgery	0.494	Unilateral	Reference	
		Bilateral	1.50	(0.46, 4.77)
Sex	0.066	Female	Reference	
		Male	0.35	(0.10, 1.07)
Breed	0.685	Non‐purebred	Reference	
		Purebred	1.25	(0.42, 3.68)
Hospitalisation at SUB placement (days)	0.037^a^		1.20	(1.01, 1.49)
Culture when SUBs placed	0.129	Negative	Reference	
		Positive	2.80	(0.74, 11.13)
*Visit history and physical examination*				
Age	0.893		0.99	(0.85, 1.15)
Time from previous flush (months)	0.166		0.81	(0.58, 1.09)
Stranguria	0.948	No	Reference	
		Present	1.04	(0.33, 3.16)
Pollakiuria	0.426	No	Reference	
		Present	1.56	(0.52, 4.66)
Haematuria	0.882	No	Reference	
		Present	1.11	(0.26, 4.22)
Lower urinary tract signs (any)	0.583	No	Reference	
		Present	1.34	(0.47, 3.93)
Body condition score	0.247		0.74	(0.43, 1.23)
*Urinalysis*				
Urine specific gravity	0.020^a^		0.89	(0.78, 0.98)
pH	0.011^a^		2.78	(1.25, 7.51)
Protein	0.084		1.57	(0.94, 2.70)
Glucose	0.270		0.56	(0.10, 1.45)
Blood	0.238		0.80	(0.55, 1.16)
Red Blood Cell Count (RBC) per high power field (HPF)	0.154	<10	Reference	
		10–20	1.75	(0.16, 20.03)
		20–50	0.17	(0.005, 2.66)
		50–100	0.71	(0.07, 7.69)
		100–250	0.17	(0.005, 2.66)
		>250	0.36	(0.03, 3.62)
WBC count per HPF	<0.001^a^	<5	Reference	
		5–50	2.54	(0.33, 52.55)
		>50	74.67	(9.91, 1658.03)
Epithelial cells (per low power field)	0.004^a^	<5	Reference	
		5–10	0.20	(0.02, 1.55)
		10–30	0.17	(0.02, 1.03)
		30–50	1.17	(0.16, 8.59)
		50–100	0.25	(0.02, 2.07)
		>100	0.03	(0.001, 0.28)
Bacteria on sediment	<0.001^a^	None	Reference	
		Present	52.00	(8.64, 1012.06)
*Blood assays*				
Creatinine (µmol/L)	0.330		1.002	(0.998, 1.007)

Multivariable analysis				
Urine pH	0.042		3.18	(1.04, 12.13)
White blood cell count (WBC) on urine sediment	<0.001	<5	Reference	
		5–50	7.74	(0.39, 817.01)
		>50	157.68	(7.71, 25,987.11)
Bacteria on urine sediment	0.005	None	Reference	
		Present	21.71	(2.33, 558.10)

^a^ Significant values.

Univariable and multivariable logistic regression model results for risk factors associated with the presence of LUTS are depicted in Table 4. Multivariable analysis identified younger age (OR 0.82, 95% CI 0.67–0.97) and being a purebred cat compared to non‐purebred (OR 6.32, 95% CI 2.00–22.74) to be independent predictors of presence of LUTS. The model was a good fit (Hosmer–Lemeshow, *p* = 0.364).

**TABLE 4:** Logistic regression outcomes for presence of lower urinary tract signs in cats with subcutaneous ureteral bypass (SUB).

Variable	*p*‐Value	Options	Odds ratio	95% confidence interval
**Univariable analysis**				
*Cat and surgery factors*				
Type of surgery	0.094	Unilateral	Reference	
		Bilateral	2.65	(0.85, 8.94)
Sex	0.757	Female	Reference	
		Male	1.18	(0.42, 3.32)
Breed	0.003^a^	Non‐purebred	Reference	
		Purebred	5.06	(1.71, 16.32)
Hospitalisation at SUB placement (days)	0.222		0.90	(0.74, 1.06)
Culture when SUBs placed	0.878	Negative	Reference	
		Positive	0.90	(0.23, 3.38)
*Visit history and physical examination*				
Age	0.046^a^		0.86	(0.72, 0.997)
Time from previous flush (months)	0.590		0.93	(0.70, 1.21)
Culture	0.583	Negative	Reference	
		Positive	1.34	(0.47, 3.93)
Body condition score	0.372		0.80	(0.49, 1.30)
Cystitis medication	0.120	None	Reference	
		Yes	4.96	(0.68, 100.36)
*Urinalysis*				
Urine specific gravity	0.479		0.97	(0.88, 1.06)
pH	0.863		1.06	(0.54, 2.11)
Protein	0.298		1.30	(0.79, 2.18)
Glucose	0.304		1.53	(0.68, 4.30)
Blood	0.357		0.85	(0.59, 1.20)
Red blood cell count per high power field (HPF)	0.680	<10	Reference	
		10–20	1.20	(0.11, 13.31)
		20–50	0.75	(0.06, 9.59)
		50–100	0.50	(0.04, 5.46)
		100–250	0.40	(0.03, 5.33)
		>250	1.38	(0.14, 13.58)
White blood cell count per HPF	0.184	<5	Reference	
		5–50	1.47	(0.40, 5.85)
		>50	3.43	(0.86, 15.25)
Epithelial cells (per low power field)	0.155	<5	Reference	
		5–10	2.63	(0.31, 27.54)
		10–30	1.75	(0.25, 15.58)
		30–50	14.00	(1.84, 172.41)
		50–100	3.50	(0.39, 39.97)
		>100	3.50	(0.61, 28.71)
Bacteria on sediment	0.608	None	Reference	
		Present	1.38	(0.40, 4.87)
Crystals	0.501	None	Reference	
		Present	0.65	(0.17, 2.25)
*Blood assays*				
Creatinine (µmol/L)	0.483		0.998	(0.99, 1.003)
Ionised calcium (mmol/L)	0.610		3.43	(0.03, 534.96)
**Multivariable analysis**				
Age at visit	0.0499		0.85	(0.70, 0.9999)
Breed	0.003	Non‐purebred	Reference	
		Purebred	5.30	(1.72, 18.19)

^a^ Significant values.

## DISCUSSION

The results of this study suggest that it is challenging to differentiate BC from SB in cats with a SUB or SUBs. Urine culture results and presence of LUTS were not significantly associated. In multivariable analysis, independent predictors of a positive urine culture were higher urine pH, marked pyuria and bacteriuria. Independent predictors of the presence of LUTS were purebred status and younger age.

The differentiation between BC and SB could not be determined in 29% of cats with positive urine cultures due to inconsistency between a positive culture and concurrent LUTS. This was likely because such a large proportion of cats (61%) showed LUTS regardless of culture result; therefore, individual cats frequently demonstrated LUTS with a negative concurrent urine culture. Transient LUTS have previously been reported in cats with SUBs,^6^, ^14^ highlighting the need for a detailed history of LUTS every time a cat with a SUB(s) is reevaluated.

In our study, 61% of cats showed LUTS at some point following SUB(s) placement, with LUTS recorded at 42% of all visits. While this incidence is high, one study found that quality of life was not affected by dysuria or gross haematuria.^5^ The incidence of LUTS is higher than in previous studies, with 3.5% dysuria,^4^ 18% dysuria and haematuria^5^ and 14% LUTS^8^ previously reported in cats with SUBs with negative urine cultures. A potential reason for this discrepancy is that effort was made to establish when LUTS were present via a history questionnaire completed by the owner at every re‐examination. This ensured owners were asked about these signs in a standardised way, whereas in other studies, this history may have not been systematically recorded and owners of cats with SUBs may have become accustomed to LUTS so failed to repeatedly mention them unless specifically asked. Additionally, a portion of these visits were during the COVID‐19 pandemic when owners may have had more time to observe their cats for these signs.

Younger and purebred cats were found to have increased odds of showing LUTS. This is in agreement with a previous study examining risk factors for feline lower urinary tract disease (FLUTD) in the wider cat population. Cats aged 4–7 years and Persian and Manx breeds were found to be at increased risk for FLUTD, while mixed breed cats were found to be at a reduced risk for congenital FLUTD.^23^

Positive urine cultures were found in 34% of cats in our study, similar to other studies on cats with SUBs, with positive cultures in 24%–36% of cats at some point during follow‐up.^4^, ^5^, ^6^, ^7^, ^8^, ^11^ One study reported that 54% of cats with SUBs had a positive urine culture at at least one timepoint, although this might have been impacted by the small sample size of 24 cats.^10^

The bacteria cultured in this study were similar to those found in other studies with *E. coli*, followed by *Enterococcus* species infections, being most common.^6^, ^7^, ^8^, ^18^, ^20^, ^23^, ^24^, ^25^ *Enterococcus* species are reported as less pathogenic,^26^ which aligns with all four cats that cultured *Enterococcus* species in this study having SB. This study also cultured *Serratia* species, *Psychrobacter faecalis* and Gram‐positive bacilli, which are rarely reported as pathogens of the lower urinary tract. It is possible that the presence of a urinary tract implant (SUB system) may make bacterial colonisation with opportunistic species more likely, although further investigation is required to confirm this.

Interestingly, hospitalisation length after surgical placement of SUB(s) was not associated with a positive culture despite all outliers for hospitalisation duration (cats with longer hospitalisation periods) subsequently culturing positive during follow‐up. This has previously been reported as a risk factor for bacteruria in some studies.^7^

Urine pH was higher in cats with a positive culture, in agreement with a previous study.^26^ Based on human studies, one contribution to this association could be that certain bacteria cultured, including *P. aeruginosa* and *Staphylococcus* species, produce urease, which splits urea to ammonia, increasing urine pH.^27^ However, most infections in this study were *E. coli*, which is not considered to increase urine pH.^28^ It is possible that acidic urine has protective functions against development of bacteriuria.^29^ Concentrated urine is also thought to be protective against infection,^29^ which may explain why a lower USG was associated with positive culture in this study and another^18^ in a univariable model.

Positive cultures were also associated with the presence of high numbers of WBCs on urine sediment. Active sediment and positive cultures were found to be associated in previous studies,^18^, ^19^ despite reports that urine sediment examination varies in value to predict positive cultures.^30^ Various studies on UTIs in cats have found a link between positive urine cultures and pyuria,^21^, ^24^, ^26^ with one study finding 67% of samples to have pyuria even if no clinical signs were documented.^18^ Haematuria is also associated with positive cultures^24^, ^26^; however, this was not found to be associated in this study and others.^18^, ^21^ This is likely because a high proportion (85%) of samples had microscopic haematuria regardless of the presence of infection, which could be due to irritation of the bladder epithelium by the cystotomy tube or bleeding during sampling (skin bleeding from the Huber needle).

Bacteria seen on sediment examination was also associated with positive cultures in this and other studies.^18^, ^24^ However, sometimes bacteria were seen despite a negative culture. This may be due to the bacteria not being viable, anaerobic or inaccurately identified.^21^ Cellular debris can be mistaken for bacteria,^31^ and RBCs were previously found to increase the probability of falsely detecting bacteria.^21^ Conversely, bacteria were not seen on sediment in some positive cultures—sensitivity can be around 81%–89% and is operator dependent.^21^

Interestingly, in this study, sex was not associated with LUTS or positive cultures despite an association between males and idiopathic cystitis^2^, ^30^ and older females and UTIs.^18^, ^19^, ^23^, ^24^, ^26^ This may be a type II error due to the small sample size or reflect the possibility that the presence of a SUB changes the risk of both a positive urine culture and LUTS in both sexes compared to cats without these devices.

The ISCAID guidelines recommend not to treat SB,^13^ due to the limited case benefit and potential for increasing antimicrobial resistance.^16^ However, these guidelines are not specific to cats with urinary tract implants. Subclinical bacteriuria is not associated with survival, development of kidney disease^32^ or progression of kidney disease in cats with chronic kidney disease (CKD).^7^, ^19^ Additionally, treatment of SB does not prevent future bacteriuria, pyelonephritis or signs of FLUTD in cats with CKD.^19^ Infection per se was not associated with survival in cats with SUBs, although no differentiation was made between SB and UTIs.^4^ However, in one study, a cat with a SUB with SB subsequently developed a perirenal abscess and severe pyelonephritis.^7^ Another study found that cats with positive cultures, either before or after SUB placement, were more likely to develop device occlusion, with *E. coli* found to be associated with device removal or replacement.^6^ These studies raise concern for the possibility of adverse outcomes if SB is not treated in cats with SUBs and highlight the need for further studies on the balance between antimicrobial stewardship to limit the development of multidrug‐resistant infections versus the possible impact of considering benign neglect for SB when managing positive urine cultures in the presence of a SUB.

Lower urinary tract signs were absent in 56% of positive cultures in this cohort, which is much higher than 7%–23% SB reported in previous studies of cats with SUBs.^7^, ^8^, ^10^, ^11^ However, these studies were smaller samples,^7^, ^10^, ^11^ only performed cultures in cats with clinical signs suggestive of UTI^7^ and/or included cats with both SUBs and ureteral stents.^11^ A large study reported 43% of positive cultures in cats with SUBs to be subclinical^6^; the study population was similar to the present cohort because it was from the same hospital, although it mostly covered a different timeframe.^6^ Furthermore, LUTS had not been systematically recorded during the time period of the aforementioned study; therefore, a detailed analysis of the presence of BC versus SB was not possible.

Given the high prevalence of positive cultures in cats with SUBs in this and previous studies,^4^, ^5^, ^6^, ^7^, ^8^, ^10^, ^11^ additional steps to prevent infection may be warranted. More studies on the frequency of SUB flushes, especially with tetrasodium EDTA (to prevent biofilm formation) and the frequency of postoperative complications, may help reduce infections and decrease complications. Additionally, the benefits of postoperative antibiotics could be considered, as one small study found that they reduced the risk of positive cultures in cats with SUBs,^11^ although this must be balanced against antimicrobial stewardship.

A limitation of this study was that clinical signs were based on owner reports and required owners to note an abnormality. Owners may not have been monitoring for these signs, especially in indoor/outdoor cats; however, the use of a questionnaire attempted to mitigate this by ensuring owners were asked about LUTS. A second limitation was the use of haematuria as an indicator of lower urinary tract disease when we were unable to confirm that the blood was not coming from the upper urinary tract. However, only two of 230 visits reported gross haematuria as the sole LUTS, with only one of these visits being used in statistical analysis; therefore, if haematuria was occurring due to upper urinary tract bleeding, this is unlikely to have impacted the results of this study. Additionally, it is possible that the proportion of positive cultures in this study may have been underestimated if bacteriuria was found and treated by a primary care veterinarian between visits to the QMHA and not reported. However, this is unlikely because primary veterinarians, or owners, usually reported such bacteriuria. As a retrospective study, there were limitations in variability and accuracy of data. Cats were managed by a variety of clinicians and clinicopathological testing was not entirely standardised. While visits with similar test runs were preferentially used, this could not always be achieved. Furthermore, not every cat had an equal number of revisits and there may have been a bias towards detecting LUTS in those that had more revisits.

## AUTHOR CONTRIBUTIONS

Rebecca Geddes conceived and designed the project. Luba Djoneva acquired, analysed and interpreted the data. All authors contributed to the revision of the article.

## CONFLICTS OF INTEREST

The authors declare they have no conflicts of interest.

## FUNDING INFORMATION

The authors received no specific funding for this work.

## ETHICS STATEMENT

The authors confirm that they adhered to the ethical policies of the journal, as noted on the journal's author guidelines page. No ethical approval was specifically required from our institutional ethics committee because this was a retrospective study.

## Supporting information

S1 Study questionnaire.
S2 Microscopic findings of urine sediment examination and corresponding R code.

## Data Availability

The data that support the findings of this study are available from the corresponding author upon reasonable request.
