# Supplementary material for: Analysis of lower urinary tract signs and bacteriuria in cats with subcutaneous ureteral bypass systems
Source: Vet Rec Open. 2023 Aug 1;10(2):e69. doi: 10.1002/vro2.69 (PMC10393289; doi:10.1002/vro2.69)
Supplement: Supplementary file 1 — S1 Study questionnaire. S2 Microscopic findings of urine sediment examination and corresponding R code. [file VRO2-10-e69-s001.docx]

**Supporting Information**

**S1 Subcutaneous Ureteral Bypass cat questionnaire**

**
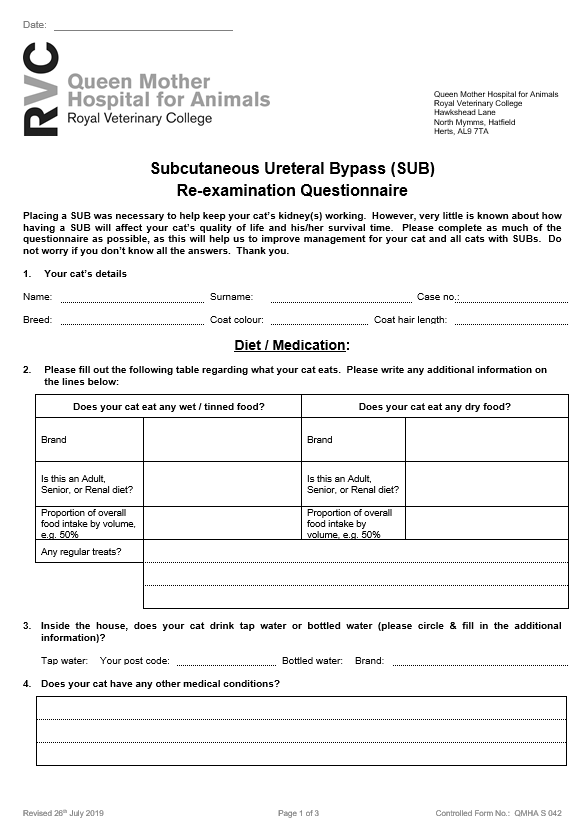
**

**
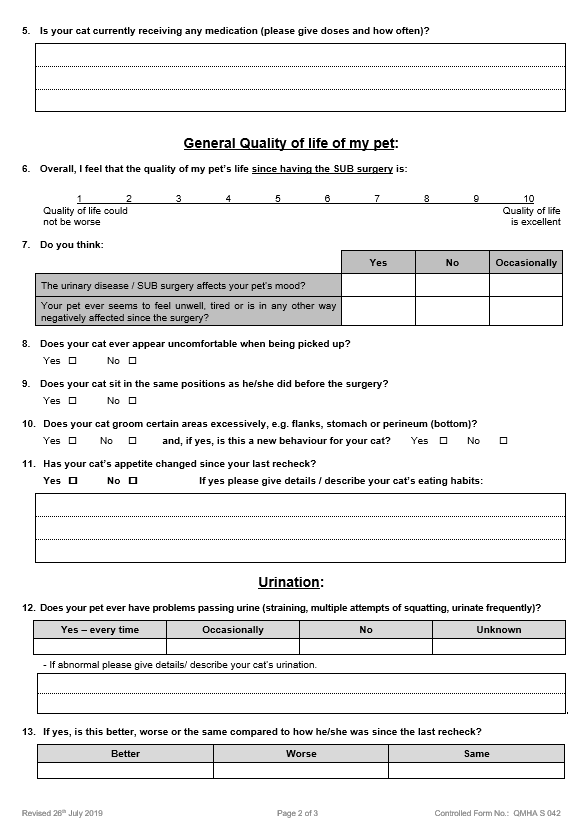
**

**
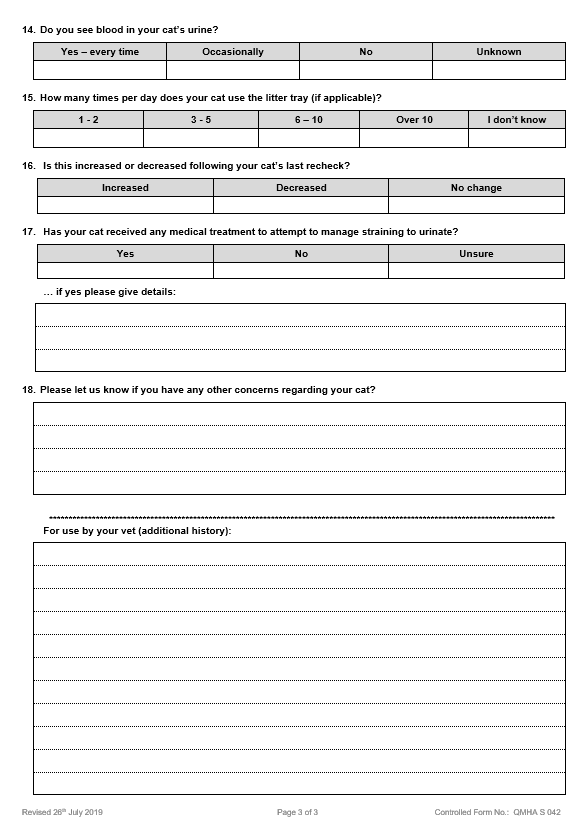
**

**S2 Microscopic findings of urine sediment examination and corresponding R code.**

|  | **Microscopic Findings** | | **R Code** |
| --- | --- | --- | --- |
|  | *Count* | *Description* |  |
| **RBC** | <10 | None | 0 |
|  | 10-20 | Few | 1 |
|  | 20-50 |  | 2 |
|  | 50-100 | Some, moderate | 3 |
|  | 100-250 | Many | 4 |
|  | 250+ | Lots, too numerous | 5 |
| **WBC** | <5 | None | 0 |
|  | 5-50 | Few, some | 1 |
|  | 50+ | Moderate, many, too numerous | 2 |
| **Epithelial cells** | <5 | None | 0 |
|  | 5-10 | Few | 1 |
|  | 10-30 | Occasional | 2 |
|  | 30-50 | Some | 3 |
|  | 50-100 | Moderate | 4 |
|  | 100+ | Many, too numerous | 5 |
